# The Late King

**Published:** 1830-10-01

**Authors:** 


### MISCELLANIES.

### XIII.

### THE LATE KING.

Now that the marble tomb has closed over the cold remains of His late Majesty, and the columns of our contemporaries, medical and popular, have ceased to pour forth their daily and hebdomadal lucubrations on the nature, cause, treatment, and probable event of a disease, the subject of which they had not seen, and the symptoms of which they were not permitted to know, we may be pardoned for making a few observations on this tragical scene of suffering and of death.

The life of irregularity—*medically* speaking, we might, perhaps, call it INTEMPERANCE—which the vigorous PRINCE and indulgent MONARCH is well known to have led, could scarcely fail to impair the functions, and, ultimately, derange the structure, of some of the vital organs. Gout was the first manifestation of this constitutional disturbance, and doubtless warded off, for a long time, more dangerous consequences—partly by the reduction of an overloaded system, through the medium of pain, depletion, and abstinence—partly by determining plethora and irritation to structures not essential to the existence of life. Pain is seldom borne with patience, even by philosophers—and still less by princes. That his late Majesty had, for some years, been in the habit of allaying the sufferings and curtailing the paroxysms of gout by full doses of WILSON'S TINCTURE, we have some reason to believe, and we need scarcely say that such bye-ways, or, as we might here call them, royal roads to temporary health, seldom fail to entail on the experimenters a train of disorders more dangerous, if not more dolorous, than those which were set up by the wise hand of Nature, but interrupted in their course by the interferences of art. Three powerful classes of causes contributed to augment and exasperate the effects of this reiterated, but perhaps unavoidable application to colchicum, for the abbreviation of the arthritic attack:—full and stimulating regimen—inactivity—and a humid, or rather malarious atmosphere. The existence of the first two classes of morbific agencies in His Majesty's case, is too well known to require proof in this place. Those who have examined the medical topography of the cottage, Virginia Water, and all those routes where the King spent so much of his time in inactive repose, or, at the most, in *passive* exercise, can only wonder that His Majesty's constitution so long resisted the usual and almost inevitable effects of an atmosphere for ever loaded with moisture, or impregnated with vegeto-animal exhalations.

We have no means of judging of the actual phenomena which the King's ordinary health may have presented, previously to the issue of the first and celebrated bulletin, respecting the "bilious attack," and "embarrassment in breathing," on the 15th of April last. It was not likely that the somewhat vague phraseology made use of, on this occasion, should escape the censure of those who, having neither responsibility nor practical knowledge themselves, delight in affixing the stigma of ignorance on others. Thus a great outcry has been raised against the King's physicians, because they did not state, in the public bulletins, the precise NAME and NATURE of a disease, which, perhaps, not one physician in Europe would have been able to have stated, with any thing like certainty —no, not even Laennec, had he been alive, and admitted to examination of the royal chest! And by whom has this weighty accusation against His Majesty's physicians been raised? By men so ignorant of practical medicine —and more especially of auscultation, that they could not tell which end of the stethoscope was proper to be applied to the ear or the body—men so ignorant of morbid anatomy, that they did not know either the name or the nature of the disease, when the minutes of the dissection were lying on the critical table before them!! This will scarcely be believed; but we pledge ourselves to prove it.

Before proceeding to this task, however, we shall make a remark or two on the moral or ethical part of the question. It is the bounden duty of the medical attendant, when he cannot arrest the progress of a mortal malady, *not to accelerate* the fatal event. No political interest—no state policy can or should bend this maxim from the direct and inflexible line of uncompromising punctuality. The prince and the peasant are on terms of rigid equality in this respect. What practical physician, then, we would ask, could be so cruel, so unfeeling—so unprofessional, as to allow a patient, labouring under organic disease of the heart, to be made acquainted with the astounding fact and fiat, that for him HOPE NO LONGER EXISTED! The genius of a NERO, a CALIGULA, a HELIOGABALUS, never invented a torture so truly diabolical as this announcement, so advocated and lauded by the self-elected HERO of HUMANITY, and a narrowing JUNTO of disappointed democrats! Every medical man, excepting those who know nothing of diseases of the heart, and those who have no hearts in their own bosoms, are well aware that such an announcement as is above alluded to would, in all probability, be attended with most imminent danger—perhaps death, to their patient. Such is the cold-blooded policy of scribbling tyrants, who, from a conscious sense of their own ignorance, would shrink from the responsibility of a diagnosis—or hesitate not to assert the most wilful falsehoods in the event of a detection of their blunders!

It is unnecessary to tell the well-informed of the profession, that no extent of experience—no amount of pathological knowledge, can enable a medical practitioner to give any thing like an accurate prognosis as to the *duration* of an organic disease of the heart, even when he is convinced of the existence of that terrible malady and of its final result. Many who labour under the most formidable symptoms linger out for months and years—while others, whose symptoms are by no means alarming, drop off suddenly, when no apprehension of death is entertained by physician, patient, or friends. Who, then, would be so ungenerous or unjust as to blame the physicians of His late Majesty, for not at once proclaiming to the nation that which no human sagacity could tell or foretel—the precise nature, duration, or degree of danger of an organic disease? None, but the ignorant or the *unprincipled!* Sorry are we to say, that the latter class is almost exclusively to be found, in this instance, among the ranks of our own profession!

The medical advisers of His late Majesty were denounced for their ignorance, in not knowing the name of the disease, and only calling it by one of its symptoms—"THE EMBARRASSMENT OF BREATHING." Yet more than half of the long black catalogue of human afflictions, with all their hard and uncouth nomenclature, are only the names of SYMPTOMS—and certain we are that the very best practitioners prescribe, in nine cases out of ten, for *symptoms alone*. When they prescribe for *names* of diseases, they are generally wrong! Then there is no such disease as "embarrassment of breathing," according to these wise-acres. What is dyspnœa—the third genus in Dr. Good's order pneumonia? What is dysphagia, but a symptom? What is Bex, (cough,) the first genus of the order pneumonia? Why a symptom; and yet it is laid down in our best nosologies as a distinct disease. Suppose the physicians had considered (and they would not have been very far wrong) the Royal Patient to be labouring under angina pectoris. The classical name of this disease is sternalgia, according to Good. Sternalgia!—" Suffocating breast-pang." What is this but a symptom?—and not a whit more indicative of the nature of the disease which it is designed to represent, than " embarrassment of breathing" was of His Majesty's disorder. But the ignorance and dishonesty of these scurrilous hyper-critics are truly disgusting, as we shall now proceed to demonstrate.

### Official Account of the Post-mortem Examination of His late Majesty.

The body exhibited but little sign of putrefaction; and the anasarca had disappeared, excepting some slight remains of it in the thighs.

Notwithstanding the apparent emaciation of His Majesty's person, a very large quantity of fat was found between the skin and the abdominal muscles.

*Abdomen.* The omentum, and all those parts in which fat is usually deposited, were excessively loaded with it. The abdomen did not contain more than an ounce of water.

The stomach and intestines were somewhat contracted; they were of a darker colour than natural, in consequence of their containing mucus tinged with blood; and in the stomach was found a clot of pure blood, weighing about six ounces.

The liver was pale, and had an unhealthy granulated appearance.

The spleen, although larger than usual, was not otherwise diseased, and the pancreas was in a sound state.

The sigmoid flexure of the large intestine (the colon) had formed unnatural adhesions to the bladder, accompanied by a solid inflammatory deposit of the size of an orange.

Upon a careful examination of this tumor, a sac or cavity was found in its centre, which contained an urinary calculus of the size of a filbert, and this cavity communicated by means of a small aperture with the interior of the bladder at its fundus. In other respects the bladder was healthy, and the prostate gland did not appear to be enlarged. The kidneys were also free from disease.

*Thorax.* Two pints of water were found in the cavity of the right side, and three pints and three quarters in the left side of the chest. The left lung was considerably diminished.

The lower edge of each lobe of the lungs had a remarkable fringe, which, upon examination, was found to be formed by a deposit of fat.

The substance of the lungs had undergone no change of structure, but the mucous membrane lining the air-tubes was of a dark colour, in consequence of its vessels being turgid with blood.

The pericardium (or heart-purse) contained about half an ounce of fluid, but its opposite surfaces in several parts adhered to each other, from inflammation at some remote period.

Upon the surface of the heart and pericardium there was a large quantity of fat, and the muscular substance of the heart was so tender as to be lacerated by the slightest force: it was much larger than natural. Its cavities upon the right side presented no unusual appearance, but those on the left side were much dilated, more especially the auricle.

The three semilunar valves at the beginning of the great artery (the aorta) were ossified throughout their substance, and the inner coat of that blood-vessel presented an irregular surface, and was in many parts ossified.

The original disease of His Majesty consisted in the ossification of the valves of the aorta, which must have existed for many years, and which, by impeding the passage of the current of blood flowing from the heart to the other parts of the body, occasioned effusion of water into the cavities of the chest and in other situations. This mechanical impediment to the circulation of the blood also sufficiently explains those other changes in the condition of the body which were connected with His Majesty's last illness, as well as all the symptoms under which the King had laboured.

The immediate cause of His Majesty's dissolution was the rupture of a blood-vessel in the stomach.

Henry Halford.
Matthew John Tierney.
Astley Paston Cooper.
B. C. Brodie.

Those who are in the habit of attending to the symptoms of diseases during life, and examining their causes or effects after death, need not be told that the ossification of the semilunar valves, in the above case, must have been of old standing—must have been in the very same condition six months ago, when His Majesty was driving about, in apparent health, as when, on the 15th April, the embarrassment of breathing was first announced. This ossification of the semilunar valves (even if the origin of the aorta was contracted, which does not appear on the record) could have had nothing to do with the sudden attack of dyspnœa. That phenomenon was clearly the consequence of the serous effusion into one or both sides of the chest. But what was the cause of this effusion? Not, we think, the ossification of the semilunar valves:—*first*, because we frequently find these valves ossified in elderly people, without any effusion of water—and *secondly*, because we still more frequently find hydrothorax without any ossification of the above-mentioned apparatus. But the disease which was clearly at the bottom of all, in this case, was the degeneration of the muscular structure of the heart, the great deposition of fat, and the *passive* dilatation of the left chambers. These diseases (in the King's case) were, no doubt, going on for months or years;—but when they arrive at a certain height, or the organ happens to be become embarrassed by the accession of any inflammatory affection of the lungs, then effusion is the usual consequence, and a new train of symptoms is developed, which often obtains a new name, without good cause. And now let us hear the *name* which the sticklers for names (Mr. Wakley and his prompters) have given to the complaint of the King.

"In what terms can we speak of the "incompetency of the medical attendants? For, with a stethoscope in "his hand, and an ear in his head, a "tyro in the profession might have discovered, in May last, the existence of "hypertrophy of the heart, and disease of the aortal valves."—*Lancet.*

Thus this censor of the medical press—this censurer, or rather slanderer of all that is respectable in the profession, is so grossly ignorant of pathology, that he calls a heart whose muscular structure was rotten—whose surface was covered with fat—and whose chambers were passively *dilated*, an *hypertrophied* heart! Such is the pseudo-pathologist who does not know ramollissement of the muscular structure and obesity from hypertrophy!!!* It is for such a state of heart that the Ex-Warden of St. Giles would have prescribed a "regulated system of depletion." Oh! yes. The Lancet is an admirable remedy for "a tender and lacerable heart." The pathologist of St. Giles's tells us that "the embarrassment in the King's breathing arose from a disordered state of the heart's action, the blood not being *propelled* with its natural regularity and velocity through the *lungs*." Indeed! A heart in a state of *hypertrophy* (as he affirms it to be in) or superbundance of muscular structure, not able to drive the blood through the lungs! True it is, that the dissectors inform us that there was *nothing unusual* in the right or pulmonary chambers of the heart—while it was the *left* chambers that were dilated, and consequently incapable of throwing the blood with vigour or velocity through the *aortic system*. But all this is Arabic to Squire Wakley, who appears to know as much about the *propelling* powers of the heart, in the morbid condition above recorded, as he does of the inhabitants of the Moon.

---

* "Quelques auteurs, et particulièrement M. Laennec, proposent de donner ce nom (hypertrophie) à l'*augmentation* d'epaisseur, et presque toujours de *consistance* de la substance *musculaire* du cœur, *sans aggrandissement* dans la capacité des ventricules et des oreillettes."—*Dict. de Sciences Med. (Nouv.) Tom. 5, p. 426.*

"The inflamed action of the membrane *covering* as well as *lining* the heart's *cavities (!!)* continued unabated; hence was laid the foundation for the watery effusion, the deposition of lymph into the pericardium, producing adhesions, the thickened irregular state of the aorta, and *ossification of its valves*."

What is this membrane which *covers* the heart's *cavities?* Where is there any mention of inflammation of the membrane lining those cavities? The membrane covering the heart's *parietes* had been inflamed at some remote period; and as for the "watery effusion into the pericardium," it amounted to *half an ounce*—an obvious post-mortem effusion. The *ossification*, too, of the semilunar valves was produced (according to the Lancet) by the same inflammation which caused the watery effusion—namely, between March and June of the present year! What a pity that Mr. Wardrop's advice was not taken on the 28th March, as it went to the effectuation of a revulsion of all this pathological condition (then considered gouty) to the lower extremities!

Such is the jumble of political pathology which the LANCET has brought forth with the aid of its prompters. A more monstrous mass of ignorance and imbecility never graced the pages of that publication—and that is the strongest term of reprobation which language can convey.

As to the political and personal malignity of this "composition of mud and blood," (an expression applied by the preceptor of TIBERIUS to that worthy prototype of the LANCET) we shall not sully our pages by any examination of them. They are disgusting and revolting monuments of the depraved appetite for vulgar slander and scurrilous abuse in a liberal and learned profession! We have exposed the astounding ignorance and utter incapacity of the medical writers in the LANCET, and this is the only function which we consider ourselves as called upon to perform.*

One reflexion may be permitted in conclusion:—Much as His late Majesty suffered, during the last six weeks or two months of his life, he narrowly and fortunately escaped years of torture. Had the calculus not become sacculated, what a series of sufferings would the King have been exposed to—and how formidable would have been the operation of lithotomy in such a subject!

Excessi e vitæ ærumnis facilesque lubensque,

Ne pejora ipsa morte dehinc videam.

* We cannot conscientiously close this subject, without stating that we have information the most unequivocal, that His Majesty's physicians were well acquainted and convinced of the existence of organic disease of the heart, in His Majesty's case, many years ago. The proof of this will shortly appear. In respect to the infamous and libellous charge made against Sir Matthew Tierney, in the LANCET, we pledge ourselves (from the most authentic sources of information) that it is *false as Hell*. There is not one word of truth in the accusation; the whole of which is a fabrication, founded in blighted ambition, and personal spleen. When a villain makes a charge against an honest man, he is called upon to prove it. The *onus probandi* unquestionably falls on the slanderer; and if he can shew no ground for his calumny, he is properly punished. It is not for the honest man to come forward and prove that he is honest—but for the traducer to shew some evidence to the contrary.

